# Surgical treatment for urinary incontinence after prostatectomy: A meta-analysis and systematic review

**DOI:** 10.1371/journal.pone.0130867

**Published:** 2017-05-03

**Authors:** Yu-Chi Chen, Pin-Hsuan Lin, Yann-Yuh Jou, Victor Chia-Hsiang Lin

**Affiliations:** 1 Department of Urology, E-Da Hospital, Kaohsiung City, Taiwan; 2 Department of Health and Beauty, Shu-Zen College of Medicine and Management, Kaohsiung City, Taiwan; 3 Taiwan Food and Drug Administration, Taipei City, Taiwan; 4 School of Medicine for International Students, I-Shou University, Kaohsiung, Taiwan; 5 Taiwanese Urological Association, Taipei, Taiwan; University of Oklahoma Health Sciences Center, UNITED STATES

## Abstract

**Background:**

This meta-analysis was designed to assess the efficacy of the male sling and artificial urinary sphincter on treating post-prostatectomy incontinence by evaluating daily pad use, cure rate, frequency of improvement in incontinence, and quality of life.

**Methods:**

Medline, Cochrane, Google Scholar, and ClinicalTrials.gov were searched (until March 31, 2014) for studies that investigated the effectiveness of artificial urinary sphincter or sling surgical treatments for prostate cancer. The primary outcome was daily pad use before and after surgery and secondary outcomes were quality of life before and after surgery, and frequency of cures (no need to use of a pad for at least 1 day) and improvements (decreased pad usage) in incontinence after surgery.

**Results:**

We found that that both the sling and artificial urinary sphincter significantly decreased the number of pads used per day by about 3 (P-values <0.001) and increased the quality of life compared with before intervention (P-values < 0.001). In addition, the cure rate and was around 60%. Intervention resulted in improvement in incontinence by about 25% (P < 0.001).

**Conclusion:**

Our findings indicate that both sling and artificial urinary sphincter interventions are effective in reducing incontinence and improving the patient’s quality of life.

## Introduction

Prostate cancer is the most common male cancer in the Western world and the second most common form of cancer death [1]. Urinary incontinence is a common complication following radical prostatectomy with a prevalence varying widely with estimates ranging from 2% to 65.5% [2–4]. Incontinence rates after prostatectomy are dependent on a number parameters including the patient’s body mass index (BMI), age, urethral length, preoperative continence status, prostatic volume, and the surgeons experience and surgical technique [5]. Urinary incontinence in men following prostrate surgery is one of the patient’s most feared post-surgery complication due to is strong social implications and its impact on quality of life [2,3,5].

Two major treatment options for male urinary incontinence are the artificial urinary sphincter and the male sling. The artificial urinary sphincter has a high success rate of about 79% for treating post-prostatectomy incontinence and is considered the gold-standard for treatment of male incontinence [2]. There is extensive evidence on the efficacy of the artificial urinary sphincter insertion as it has been available for a longer time than the male slings with follow-up periods ranging from 3 to 7.7 years [2]. The percentage of patients who experience treatment success (0–1 pad/day) after receiving an artificial urinary sphincter ranges from 58% to 90% [2,6]. Potential complications include incontinence (due to poor compliance in neurogenic bladders), urethral atrophy, mechanical failure, device erosion, and infection [6].

The male sling was introduced to treat men with low volume incontinence (use of 1–3 pads/day) [2]. The sling contains no mechanical components and therefore reduces the possibility of device failure. There are several different types of male slings that are used for treating post-prostatectomy incontinence including the bone-anchored slings (BAS), retrourethral transobturator (RTS), adjustable retropubic sling (ARS), and the quadratic sling [2]. The range of success for these different slings are from 40%-90% [2,6]. Common adverse events depending upon the sling are retention, infection, and perineal pain [6].

Although there are multiple studies that have evaluated the efficacy of various male sling techniques and artificial urinary sphincter insertion, the interpretation and comparison of findings between studies can be difficult [2,4]. Currently, there is no standard for reporting pre- and post-operative degrees of incontinence or a consistent way of defining success. Many of the studies have poorly defined inclusion and exclusion criteria including the definition of incontinence. Moreover, many studies include patients with incontinence due to diverse etiologies. This meta-analysis was designed to assess the efficacy of the male sling and artificial urinary sphincter by evaluating daily pad use, cure rate, frequency of patient improvement, and quality of life.

## Material and methods

### Search strategy

The systematic review and meta-analysis were conducted in accordance with the PRISMA guidelines [7]. Medline, Cochrane, Google Scholar, and ClinicalTrials.gov were searched for studies (until March 31, 2014) that investigated the effectiveness of artificial urinary sphincter or sling surgical treatments for incontinence following prostate cancer surgery. The search used the following terms: urinary incontinence, prostatectomy, sling/suspension, artificial urinary sphincter. The list of potential studies was hand searched by YCC and the data were extracted by two independent reviewers, PHL and CHL, and for any disagreement a third reviewer was consulted., CHL for the search and YCC for the data extraction.

Included studies were randomized controlled or prospective studies in patients that had undergone radical prostatectomy with subsequent complication of urinary incontinence. All included studies evaluated the efficacy of artificial urinary sphincter or sling suspension. Only publications in English were included. Studies were excluded if they were retrospective in design, the intervention for urinary incontinence was combined with prostatectomy, or the study did not quantitatively report outcomes of interest. Letters, comments, editorials, and case reports were excluded.

### Data extraction and quality assessment

The following information was extracted from studies that met the inclusion criteria: the name of the first author, year of publication, study design, demographic data of subjects, type of intervention, length of follow-up, and outcomes before and after intervention.

The quality of the included studies was evaluated using the Modified 18-items Delphi checklist [8]. This tool is designed for evaluating the quality of non-comparative study. The assessment of quality was also performed by two independent reviewers, YYJ and PHL, and a third reviewer, YCC, was consulted for any disagreements.

### Statistical analysis

The primary outcome for this meta-analysis was daily pad use before and after surgery and secondary outcomes were quality of life before and after surgery, and the percentage of cured patients (no need to use a pad for at least 1 day) and frequency of patient improvement (decreased pad usage) after surgery. For daily pad use and quality of life, mean with standard deviations were calculated and were compared between patients before and after surgery. However, if the study did not report mean and standard deviation, median, range, and the size of a sample were used to estimate the mean and variance [9]. If the median and interquartile range (IQR) were reported in a study, we assumed that the median of the outcome variable was equal to the mean response and that the width of the interquartile range was approximately 1.35 standard deviations [10]. Because quality of life was determined by various instruments, standardized difference (std diff) in means with corresponding 95% confidence intervals (CIs: lower and upper limits) were calculated for each individual study and for the pooled studies. Difference in means with 95% CI were calculated for daily pad amount and event rates and 95% CI were calculated for binary outcomes for each individual study and for the studies combined. All estimates and their 95% CI were estimated under the generic inverse variance approach. A χ^2^-based test of homogeneity was performed and the inconsistency index (I^2^) and Q statistics were determined. If I^2^ was > 50% or > 75%, the trials were considered to be heterogeneous or highly heterogeneous, respectively. If I^2^ was < 25%, the studies were considered to be homogeneous. If the I^2^ statistic were > 50%, a random-effects model (DerSimonian–Laird method) was used. Otherwise, fixed-effects models (Mantel-Haenszel method) were employed. Combined effects were calculated and a two-sided P value <0.05 was considered to indicate statistical significance. Sensitivity analysis was carried out for the primary outcome using the leave one-out approach. Publication bias was assessed by constructing funnel plots for the primary outcome by Egger’s test. The absence of publication bias was indicated by the data points forming a symmetric funnel-shaped distribution and one-tailed significance level P >0.05 (Egger’s test). All analyses were performed using Comprehensive Meta-Analysis statistical software, version 2.0 (Biostat, Englewood, NJ, USA).

## Results

**Fig 1:**
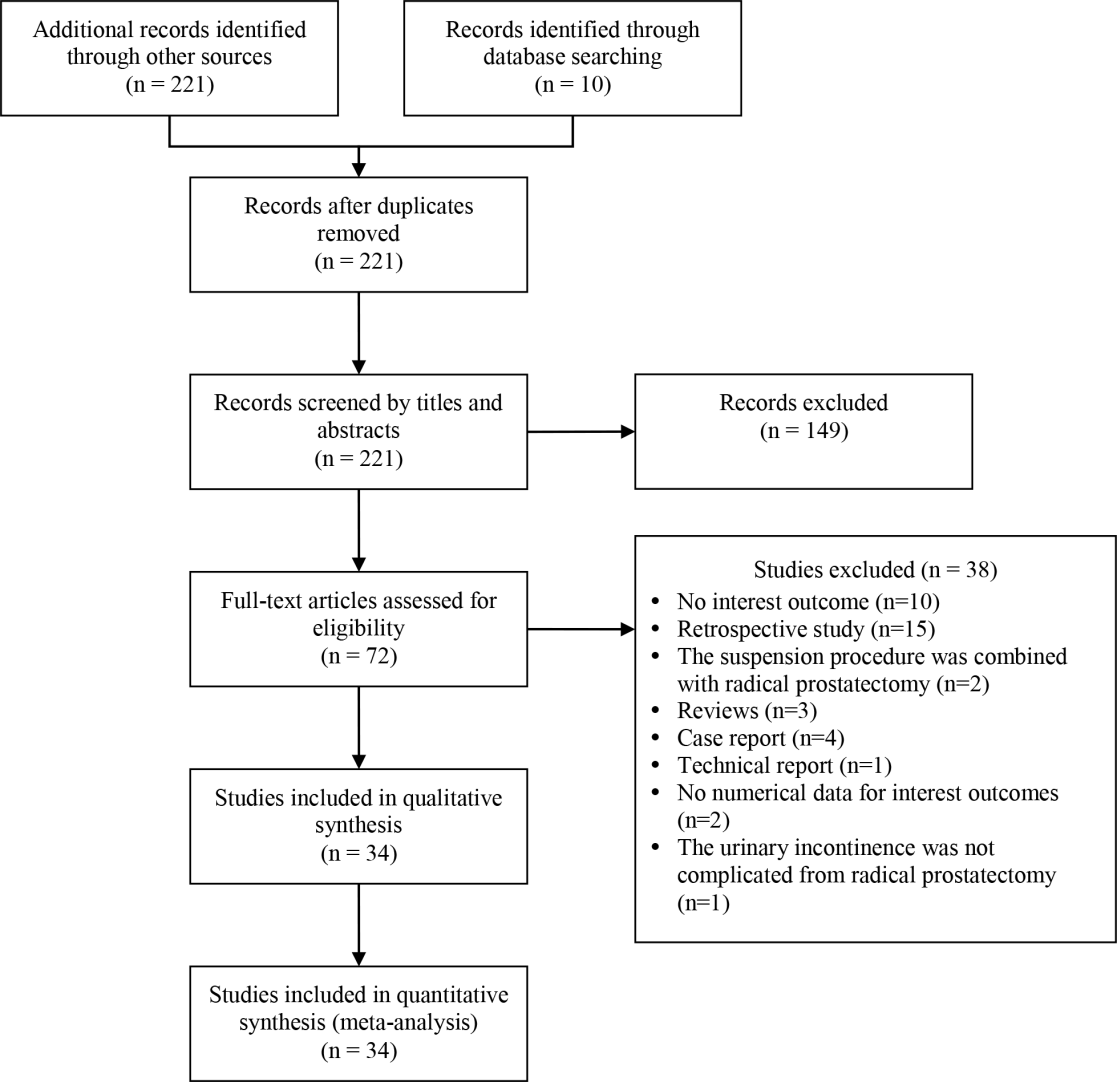
Study selection flow chart.

The initial search identified 221 articles, 149 of which were excluded upon the initial screening leaving 72 studies for full review (Fig 1). Of the 72, 38 were excluded due to not reporting an outcome of interest (n = 10), being a retrospective study (n = 15), the suspension procedure was done with the radical prostatectomy (n = 2), or was a review (n = 3), case (n = 4), or technical report (n = 1). Three studies were excluded for not reporting the findings quantitatively or the incontinence was not a result of the radical prostatectomy.

Thirty-four prospective studies, including 1 randomized control trial, were included in the analysis (Fig 1 and Table 1) [11–44]. The studies comprised 1859 patients: 1385 (n = 26 studies) received the sling procedure and 474 (n = 8 studies) were used the artificial urinary sphincter (Table 1). The mean age ranged from 64.6 to 74 years for patients treated with the sling and from 64 to 72 years for patients treated with the artificial urinary sphincter.

**Table 1:** Summary of basic characteristics and outcomes of selected studies for meta-analysis.

Study number	1at Author (year)	Number of patients	Age (years)	Duration of follow up (months)	Pad use (pads/ day) Pre vs. 1 year after surgery	Definition of cured rate	Cured rate (%)	Definition of improved rate	Improved rate (%)	QoL Instrument	QoL (Pre vs. Post)
**Sling**											
1	Grise (2012)	122	69.4	NA	2.4g. 0.6	NA	NA	Decreases pad use	87%	NA	NA
2	Leruth (2012)	173	67.3	28 (12, 60)^1^	NA	No pad use	49%	Two or fewer pads and a reduction of pads ≥50%	35%	Ditrovie quality of life	32 (14) vs. 19 (10)
3	Rehder (2012)	156	68 (63, 72)^2^	40.1 (6.0)	4.0 (2, 6)^2^ vs. 1 (0, 2.5)^2^	No pad or one dry pad for security reasons	53.8%	One to two wet pads or a reduction of pads ≥50%	23.1%	Incontinence Quality of Life	61 (45, 71)^2^ vs. 93 (72, 105)^2^
4	Bauer (2011)	24	71 (62, 77)^1^	18.8 (12, 33)^1^	NA	No pad or one dry pad for security reasons	25%	One to two wet pads or a reduction of pads ≥50%	25%	Incontinence Quality of Life	52.5 (35, 67)^2^ vs. 72 (48, 98)^2^
5	Ceresoli (2010)	12	72	26 (24, 27)^1^	NA	Complete response	58.3%	Partial response	33.3%	NA	NA
6	Cornel (2010)	35	68.5 (55.0, 82.6)^1^	NA	NA	NA	9%	NA	45.5%	NA	NA
7	Cornu (2010)	136	67.4 (6.8)	21 (6)	2.1 (1.2) vs. 0.6 (1.0)	No pad usage	62%	A decrease in pad use by > 50%	16%	NA	NA
8	Soljanik (2010)	35	68.4 (6.8)	16.6	4.4 (3.0) vs. 0.9 (3.0)	No pad or one dry pad for security reasons	72.4%	One to two wet pads or a bFreduction of pads ≥50%	17.2%	Incontinence Quality of Life	60.6 (16.9) vs. 88.3 (17.8)
9	Wadie (2010)	40	66 (20, 80)^3^	24	NA	Dry	85%	NA	NA	NA	NA
10	Bauer (2009)	124	68.9 (54, 87)^1^	NA	4 (3, 6)^2^ vs. 0 (0, 1)^2^	No pad or one dry pad for security reasons	51.4%	One to two wet pads or a reduction of pads ≥50%	25.7%	Incontinence Quality of Life	59.5 (47, 70)^2^ vs. 100 (89, 109)^2^
11	Grise (2009)	50	72 (64, 77)^2^	NA	2 (2, 3)^2^ vs.1 (0, 2)^2^	NA	NA	NA	NA	SF-36	100 (83, 133)^2^ vs. 300 (167, 375)^2^
12	Guimarães (2009)	62	69 (57, 78)^1^	28		Stopped wearing continence pads	63%	Reduction of pads ≥50%	24%	NA	NA
13	Gilling (2008)	37	69.9 (59, 79)^1^	51.5 (24, 60)^1^	2.8 (2.1) vs. 0.8 (0.9)	NA	NA	NA	NA	Incontinence Quality of Life	49.7 (19.3) vs. 81.4 (15.3)
14	Inci (2008)	19	67.5 (59, 80)^1^	17.3 (12, 25)^1^	10.3 (2.5) vs. 0.6 (1.4)	Completely dry	78.9%	Improved significantly to 1 to 2 pads per day	10.5%	NA	NA
15	Fischer (2007)	62	51 (45, 84)^1^	15 (3, 37)^1^	NA	NA	NA	Determined by the Patient Global Impression of Improvement	58%	NA	NA
16	Gallagher (2007)	31	66 (54, 83)^1^	15 (9, 21)^1^	3.7 (1, 12)^1^ vs.1.3 (0, 8)^1^	Dry or using less than 1 pad/day	75%	NA	NA	Male Urinary Symptom Impact Questionnaire	29.9 (18.8) vs.14.6 (16.1)
17	Rehder (2007)	20	65.3 (47, 81)^1^	NA	5.5 (5, 7)^1^ vs. 3.3 (3, 4)^1^	No pad usage	40%	One to two pads per day	30%	NA	NA
18	Sousa-Escando´n (2007)	51	69 (58, 81)^3^	32 (16, 50)^1^	NA	No pads or small pads or sanitary napkins for security but normally remained dry	64.7%	Important improvement	19.6%	NA	NA
19	Wadie (2007)	23	64.6 (8.9)	9 (6, 24)^3^	NA	Completely dry	87%	Greatly improved	20%	NA	NA
20	Romano (2006)	48	67.7 (52, 77)^3^	7.5 (1, 17.5)^3^	5 (3, 8)^3^ vs. NA	Dry	73%	Mild, sporadic incontinence, one or fewer pads/day	10%	Incontinence Questionnaire, Short-Form	19.2 (12, 21)^1^ vs. 4 (0, 21)^1^
21	Stern (2005)	9	74 (59, 86)^1^	48 (3.2, 79)^1^	NA	No pad	11%	One to two pads	56%	Incontinence Quality of Life questionnaires	100% (4 responses having a minimal or mild impact on their lives on urinary symptoms
22	John (2004)	19	67 (56, 83)^1^	NA	7 (2–12)^1^ vs.1 (0, 10)^1^	No pad or one dry pad	69%	Reduction of urine leakage or pads ≥50%	6%	Quality of life of incontinent men	6 (4, 6)^3^ vs. 1 (0, 5)^3^
23	Schaal (2004)	30	68 (50, 78)^3^	4 (2, 12)^3^	NA	No need for pads	67%	Requiring 1 to 2 pads daily	13.3%	NA	NA
24	Comiter (2002)	21	67 (32, 80)^1^	12 (5, 21)^1^	NA	Leakage no problem, no pads	76%	Leakage very small or small problem, 1 pad daily	14%	NA	NA
25	Madjar (2001)	16	67 (56, 74)^1^	12.2 (4, 20)^1^	NA	No or 1 pad used daily for security without any episode of leakage	75%	A decrease of 50% or more in pads daily	12.5%	NA	NA
26	Jorion (1997)	30	65 (53, 75)^1^	NA	NA	No protection needed at any time	98%	NA	NA	NA	NA
**Artificial urinary sphincter**											
1	Lai (2011)	129	69.0 (0.6)	34.1 (2.7)	5.2 (0.3) vs. 1.1 (0.1)	NA	NA	NA	NA	NA	NA
2	Hübner (2007)	50	72 (62, 80)^1^	NA	6.3 (4.3) vs. 2.1 (2.1)	0–1 security pad/d	52%	a reduction of pads ≥50%	8%	Incontinence Quality of Life	33 (19.8) vs. 64 (24.7)
3	Kocjancic (2007)	65	65.4 (25, 79)^1^	19.5 (12, 62)^1^	5.2 vs. 1.5 (3.0)	0–1 safety pad/day	67%	≥2 pads/day but >50% pad reduction	17%	Incontinence Quality of Life	31.7 vs. 71.1 (23.9)
4	Trigo-Rocha (2006)	25	68.6 (61, 72)^1^	22.4 (6, 48)^1^	4.8 (1.7) vs. 1.8 (1.6)	Using 0 to 1 pad daily and satisfied	60%	Improved but unsatisfied	12%	Incontinence Quality of Life	63.0 (20.4) vs. 82.6 (12.2)
5	Imamoglu (2005)	11	64 (52, 76)^1^	NA	1.33 vs. 0.09	Dry	90.9%	Socially continent	9.1%	SEAPI QMM	26.8 vs. 6.8
6	Kuznetsov (2000)	41	NA	19	NA	No p	29%	≤1 pad/day	37%	NA	NA
7	Mottet (1998)	103	NA	NA	NA	Dry	57%	Social continence	26%	NA	NA
8	Litwiller (1996)	50	71 (51, 83)^1^	23.4	NA	NA	NA	NA	72%	NA	NA

^1^mean (range).

^2^ median (IQR).

^3^ median (range).

NA, not available.

For patients who received the sling, the duration of follow-up ranged from 4 to 51.5 months and in all studies there was a numerical reduction in the number of pads used per day following surgery (Table 1). The cure rate (defined differently across studies) ranged from 9% to 98% and the frequency of improvement ranged from 6% to 87%. For patients treated with the artificial urinary sphincter follow up ranged from 19 to 34.1 months (Table 1). Similar to sling treatment, the number of pads used per day decreased following surgery and improvement ranged from 8% to 72%. Overall, quality of life, generally reported as impact of incontinence, following either sling of artificial urinary sphincter intervention improved following treatment (Table 1)

### Quality assessment of included studies

The results of quality assessment were summarized in Table 2. Using the criteria that a study with 14 or more yes responses out of 18 (≥ 70%) of the Delphi checklist was considered to be of acceptable quality [45], 13/26 studies that evaluated the efficacy of the sling were not of acceptable quality [13,15,17,22–24,28,29,31–35]. None of the studies that assessed the artificial urinary sphincter had ≥14 yes responses indicating they were not of acceptable quality. Criteria that were lacking in studies included the reporting of additional interventions, many studies were not multi-center, patients were not recruited consecutively, there was no report of the number of subjects lost to follow up, and competing interests and source of funding support were not reported (Table 2).

**Table 2:** The summarized results of quality assessment for included studies.

Study no.	1^st^ author (year)	1	2	3	4	5	6	7	8	9	10	11	12	13	14	15	16	17	18
		Is the hypothesis/aim/objective of the study clearly stated in the abstract, introduction, or methods section?	Are the characteristics of the participants included in the study described?	Were the cases collected in more than one centre?	Are the eligibility criteria (inclusion and exclusion criteria) to entry the study explicit and appropriate?	Were participants recruited consecutively?	Did participants enter the study at a similar point in the disease?	Was the intervention clearly described in the study?	Were additional interventions (co-interventions) clearly reported in the study?	Are the outcome measures clearly defined in the introduction or methods section?	Were relevant outcomes appropriately measured with objective and/or subjective methods?	Were outcomes measured before and after intervention?	Were the statistical tests used to assess the relevant outcomes appropriate?	Was the length of follow-up reported?	Was the loss to follow-up reported?	Does the study provide estimates of the random variability in the data analysis of relevant outcomes?	Are adverse events reported?	Are the conclusions of the study supported by results?	Are both competing interest and source of support for the study reported?
**Sling**																			
1	Grise (2012)	Y	Y	Y	Y	N	Y	Y	N	Y	Y	Y	N	Y	Y	Y	Y	Y	Y
2	Leruth (2012)	Y	Y	N	Y	Y	Y	N	N	Y	Y	Y	Y	Y	Y	Y	Y	Y	Y
3	Rehder (2012)	Y	Y	Y	Y	Y	Y	Y	N	Y	Y	Y	Y	Y	Y	Y	Y	Y	Y
4	Bauer (2011)	Y	Y	N	Y	Y	Y	Y	N	Y	Y	Y	Y	Y	N	Y	Y	Y	Y
5	Ceresoli (2010)	Y	Y	N	Y	Y	Y	Y	N	Y	Y	N	Y	Y	N	Y	Y	Y	N
6	Cornel (2010)	Y	Y	Y	Y	N	Y	Y	N	Y	Y	Y	Y	Y	Y	Y	N	Y	Y
7	Cornu (2010)	Y	Y	N	N	Y	Y	Y	N	Y	Y	Y	N	Y	N	Y	Y	Y	N
8	Soljanik (2010)	Y	Y	N	Y	Y	Y	Y	N	Y	Y	Y	Y	Y	Y	Y	Y	Y	Y
9	Wadie (2010)	Y	Y	N	Y	N	Y	Y	N	Y	Y	Y	N	Y	Y	Y	Y	Y	Y
10	Bauer (2009)	Y	Y	N	Y	Y	Y	Y	N	Y	Y	Y	Y	Y	Y	Y	Y	Y	Y
11	Grise (2009)	Y	Y	Y	Y	N	Y	Y	N	Y	Y	Y	Y	Y	N	Y	N	Y	Y
12	Guimarães (2009)	Y	Y	N	Y	N	Y	Y	N	Y	Y	N	Y	Y	Y	Y	Y	Y	N
13	Gilling (2008)	Y	Y	N	Y	N	Y	Y	N	N	N	Y	NA	Y	Y	Y	Y	Y	N
14	Inci (2008)	Y	Y	N	N	Y	Y	Y	N	Y	Y	Y	Y	Y	N	Y	Y	Y	N
15	Fischer (2007)	Y	Y	N	Y	N	Y	Y	N	Y	Y	Y	Y	Y	N	Y	Y	Y	Y
16	Gallagher (2007)	Y	Y	N	N	Y	Y	Y	N	Y	Y	Y	Y	Y	Y	Y	Y	Y	N
17	Rehder (2007)	Y	Y	N	Y	N	Y	Y	N	Y	Y	Y	NA	Y	N	Y	Y	Y	Y
18	Sousa-Escando´n (2007)	Y	Y	Y	Y	N	Y	Y	N	Y	Y	Y	NA	Y	N	Y	Y	Y	N
19	Wadie (2007)	Y	Y	N	N	N	Y	Y	N	N	Y	N	NA	Y	N	Y	N	Y	N
20	Romano (2006)	Y	Y	Y	Y	N	Y	Y	N	Y	Y	Y	NA	Y	N	Y	Y	Y	Y
21	Stern (2005)	Y	Y	N	N	N	Y	Y	N	Y	Y	Y	NA	Y	Y	Y	N	Y	Y
22	John (2004)	Y	Y	Y	N	Y	Y	Y	N	Y	Y	Y	NA	Y	N	Y	N	Y	N
23	Schaal (2004)	Y	Y	N	Y	N	Y	Y	N	N	N	N	NA	Y	N	Y	N	Y	N
24	Comiter (2002)	Y	Y	N	N	N	Y	Y	N	Y	Y	Y	NA	Y	N	Y	Y	N	Y
25	Madjar (2001)	Y	Y	N	N	N	Y	Y	N	Y	Y	Y	NA	Y	N	Y	N	Y	Y
26	Jorion (1997)	Y	Y	N	Y	Y	Y	Y	N	Y	Y	Y	Y	Y	N	Y	Y	Y	N
**Artificial urinary sphincter**																			
1	Lai (2011)	Y	Y	N	Y	Y	Y	Y	N	N	NA	Y	Y	Y	N	Y	N	Y	Y
2	Hübner (2007)	Y	Y	N	Y	N	Y	Y	N	Y	Y	Y	Y	Y	N	Y	Y	Y	N
3	Kocjancic (2007)	Y	Y	N	Y	N	Y	Y	N	Y	Y	Y	Y	Y	N	Y	Y	Y	N
4	Trigo-Rocha (2006)	Y	Y	N	Y	N	Y	Y	N	Y	Y	Y	Y	Y	N	Y	Y	Y	N
5	Imamoglu (2005)	Y	Y	N	Y	N	Y	Y	N	Y	Y	Y	N	Y	N	Y	Y	Y	N
6	Kuznetsov (2000)	Y	Y	N	Y	Y	Y	Y	N	Y	Y	Y	Y	Y	N	Y	Y	Y	N
7	Mottet (1998)	N	Y	Y	Y	N	Y	Y	N	Y	Y	Y	Y	Y	N	Y	Y	Y	N
8	Litwiller (1996)	Y	Y	N	Y	N	Y	Y	N	Y	Y	Y	Y	Y	N	Y	Y	Y	N

Y: yes; N: No; NA: not available.

### Meta-analysis of daily pad use

**Fig 2:**
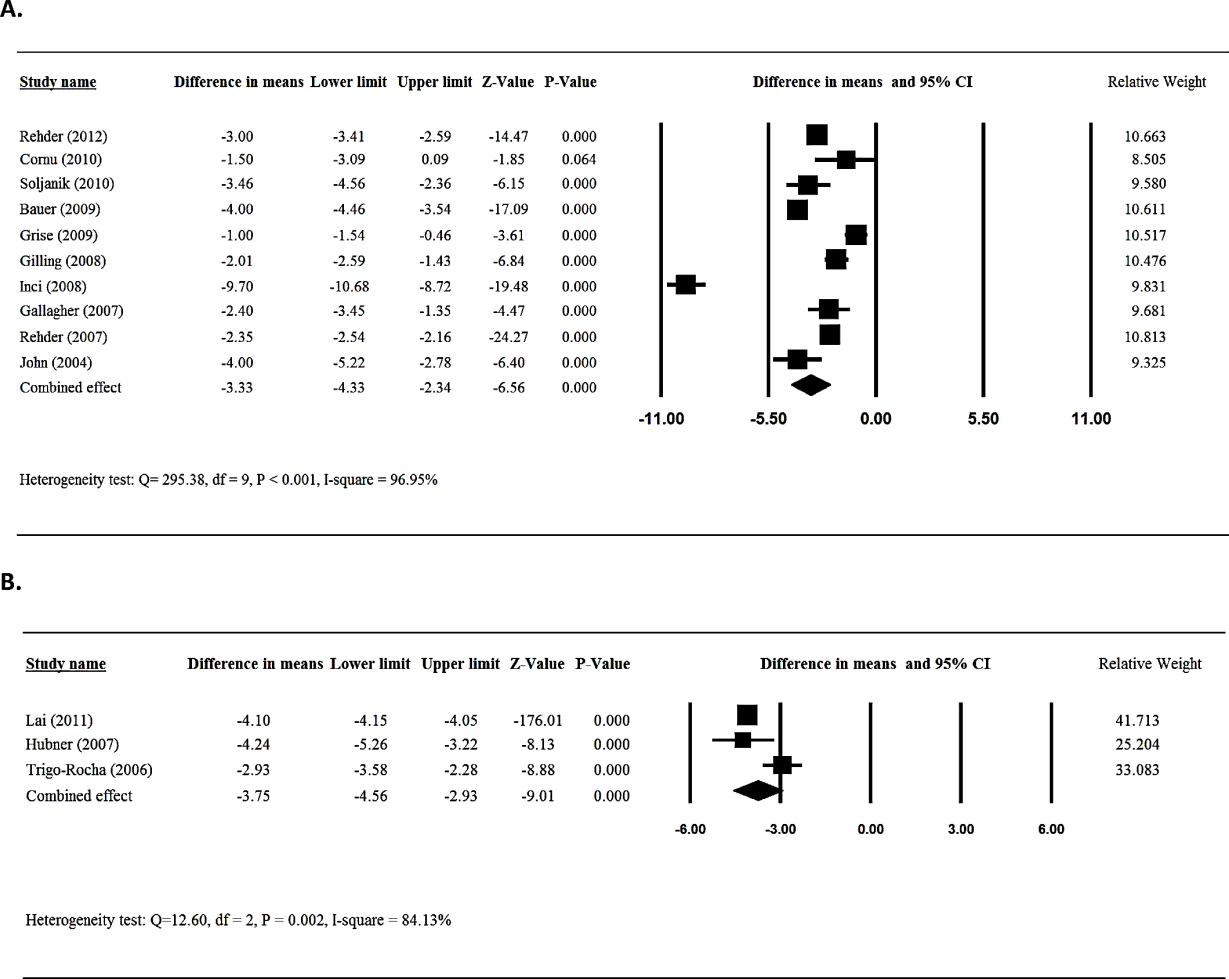
Meta-analysis for daily pad amount for patients treated with sling (A) and AUS (B).

Ten [13,17,18,20,21,23,24,26,27,32] of the 26 studies provided numerical data for patients treated with sling surgery in the daily pad use between patients before and after surgery and were included in the meta-analysis. There was evidence of heterogeneity among the 10 studies (Q statistic = 295.38, I^2^ = 96.95, P < 0.001); therefore, a random-effects model of analysis was used. The combined difference in means (-3.33 95%CI = -4.33 to -2.34) indicated that patients who received sling had a significant decrease in daily pad use following surgery (*P*< 0.001) (Fig 2A).

Three [37,38,40] of the 8 studies that reported findings pre-and post-artificial urinary sphincter implantation provided numerical data for daily pad use and were included in the meta-analysis. Heterogeneity was observed among the 3 studies (Q statistic = 12.60, I^2^ = 84.13, P = 0.002); therefore, a random-effects model of analysis was used. The results indicated that patients who received artificial urinary sphincter implantation had a significantly decreased daily pad usage post-surgery (-3.75 95%CI = -4.56 to -2.93, *P*< 0.001) (Fig 2B).

### Meta-analysis of cure rate

**Fig 3:**
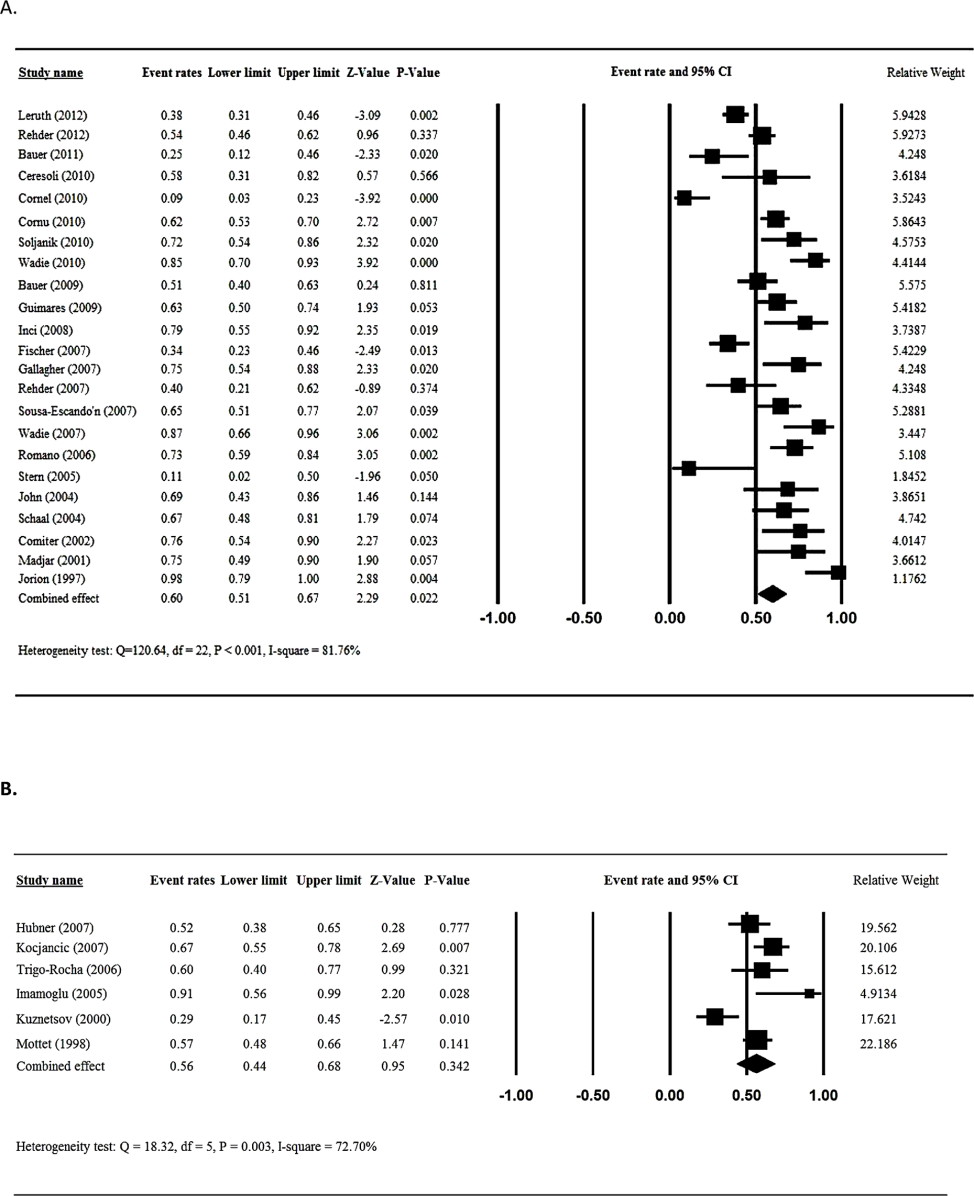
Meta-analysis for cured rate for patients treated with sling (A) and AUS (B).

Twenty-three [12–18,20,22,24–36] of the included studies reported the cure rates after male sling placement and were included in the meta-analysis. Analysis of homogeneity indicated there was heterogeneity among the 23 studies (Q statistic = 120.64, I^2^ = 81.76%, P < 0.001); hence, a random effects analysis was used. The overall cure rate for the patients after male sling placement was 60% (95%CI = 0.51 to 0.67, P = 0.022) (Fig 3A).

Six studies [38–43] that evaluated the efficacy of artificial urinary sphincter implantation and reported the cure rates following placement were included in the meta-analysis. Heterogeneity across the studies was seen (Q statistic = 18.32, I^2^ = 72.7%, P = 0.003); therefore, a random-effects analysis was used. The overall cure rate for the patients artificial urinary sphincter implantation was 56% (95%CI = 0.44 to 0.68, P = 0.342) (Fig 3B).

### Meta-analysis of improvement in incontinence

**Fig 4:**
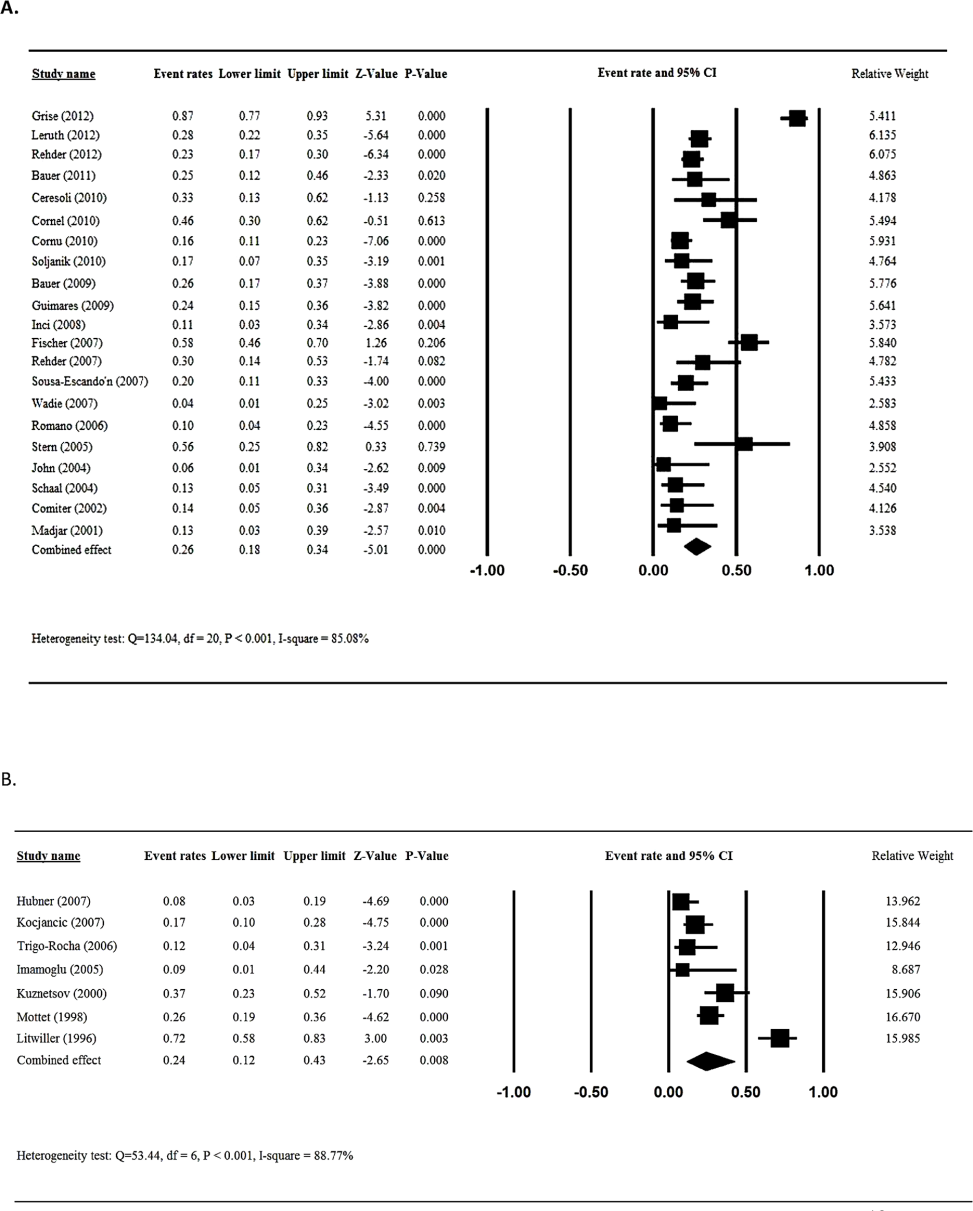
Meta-analysis for improve rate for patients treated with sling (A) and AUS (B).

Twenty-one of the included studies [11–18,20,22,24,25,27–35] reported frequency of improvement in incontinence after male sling placement and were included in the meta-analysis. Test for heterogeneity indicated there was heterogeneity across the 21 studies (Q statistic = 134.04, I^2^ = 85.08%, P < 0.001); hence, a random-effects analysis was used. The overall the frequency of patients who reported improvement in incontinence after male sling placement was 26% (95%CI = 0.18 to 0.34, P < 0.001, Fig 4A).

For the patients after artificial urinary sphincter implantation, seven studies [38–44] reported the improvement in incontinence and were included in the meta-analysis. Evaluation of homogeneity indicated there was heterogeneity among the six studies (Q statistic = 53.44, I^2^ = 88.77%, P < 0.001); consequently, a random-effects analysis was used. The overall improvement rate in incontinence for patients who received artificial urinary sphincter implantation was 24% (95%CI = 0.12 to 0.43, P = 0.008) (Fig 4B).

### Meta-analysis of quality of life

**Fig 5:**
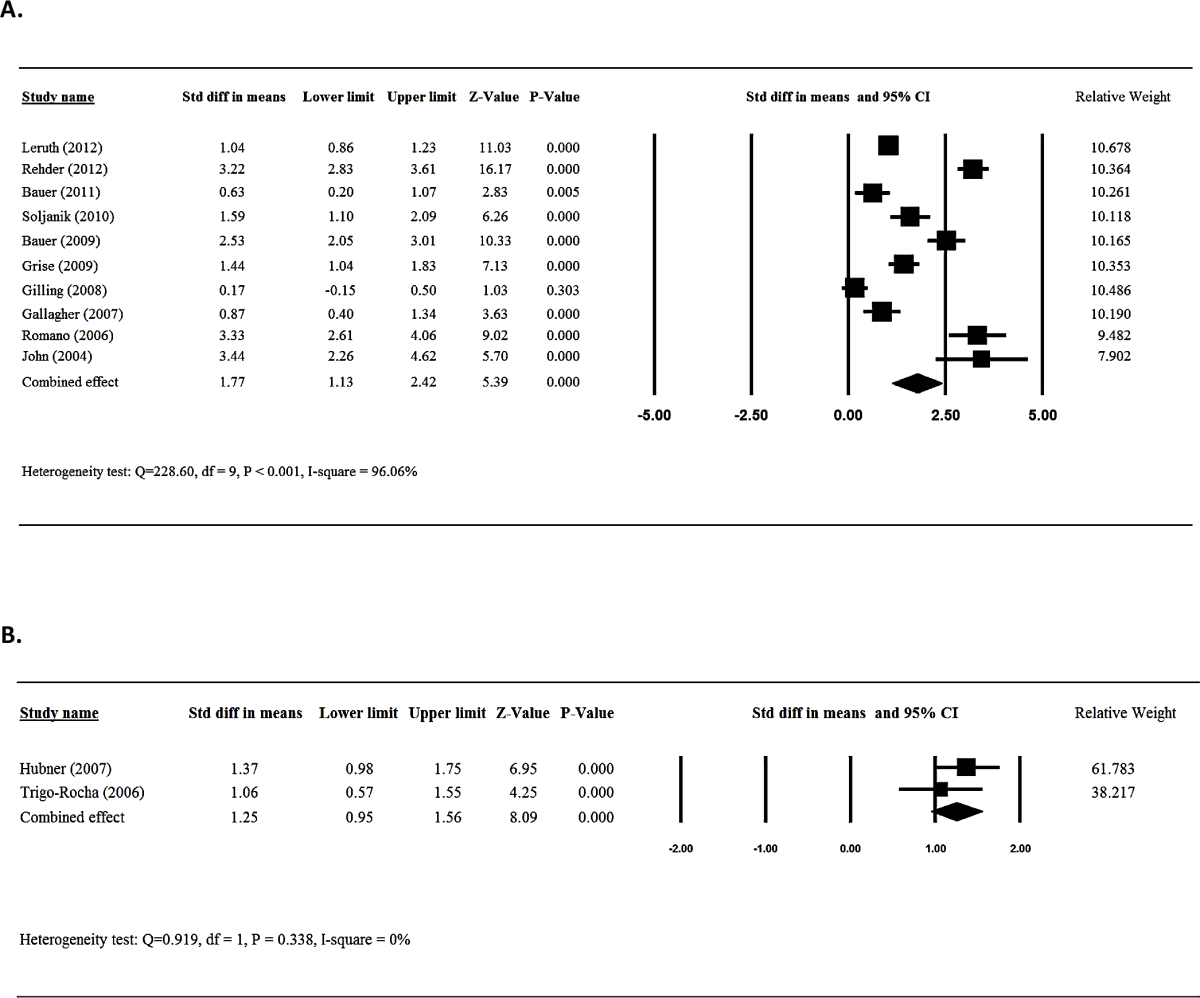
Meta-analysis for QOL score for patients treated with sling (A) and AUS (B).

Ten [12–14,18,20,21,23,26,30,32] of the 26 studies who investigated sling surgery provided numerical data for quality of life for patients before and after surgery and were included in the meta-analysis. There was evidence of heterogeneity among the 10 studies (Q statistic = 228.60, I^2^ = 96.06, P < 0.001); therefore, a random-effects model of analysis was used. Because quality of life was determined by various instruments, standardized difference (std diff) in means with corresponding 95% CIs were used. The combined std diff in means (1.77, 95%CI = 1.13 to 2.42) indicated that sling placement significantly improved a patient’s quality of life (P< 0.001) (Fig 5A).

Only two [38,40] of the eight studies that assessed the efficacy of urinary sphincter implantation provided numerical data for the quality of life for patients before and after surgery and were included in the meta-analysis. There was evidence of homogeneity for both studies (Q statistic = 0.919, I^2^ = 0, P = 0.338); therefore, a fix-effects model of analysis was used. The results indicated that patients who received artificial urinary sphincter implantation had a significantly improved quality of life after surgery (1.25, 95%CI = 0.95 to 1.56, P < 0.001) (Fig 5B).

### Sensitivity analysis and publication bias

**Fig 6:**
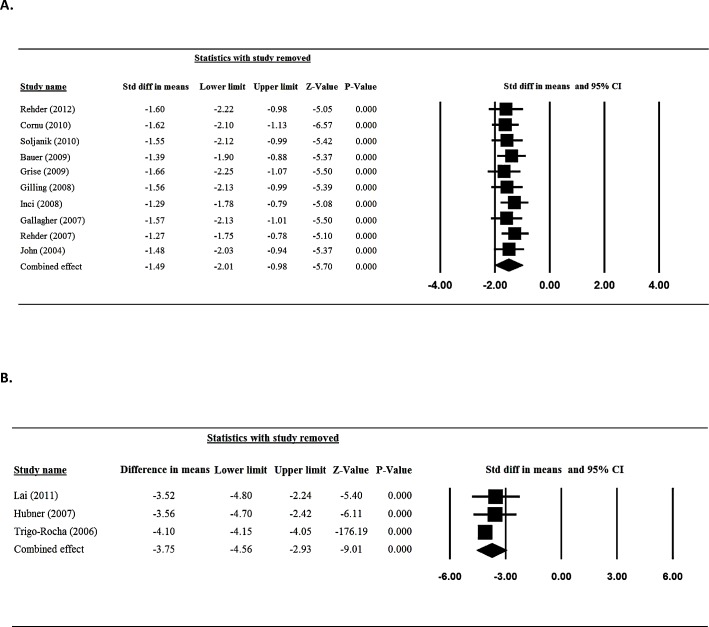
Sensitivity -analysis for daily pad amount for patients treated with sling (A) and AUS (B).

**Fig 7:**
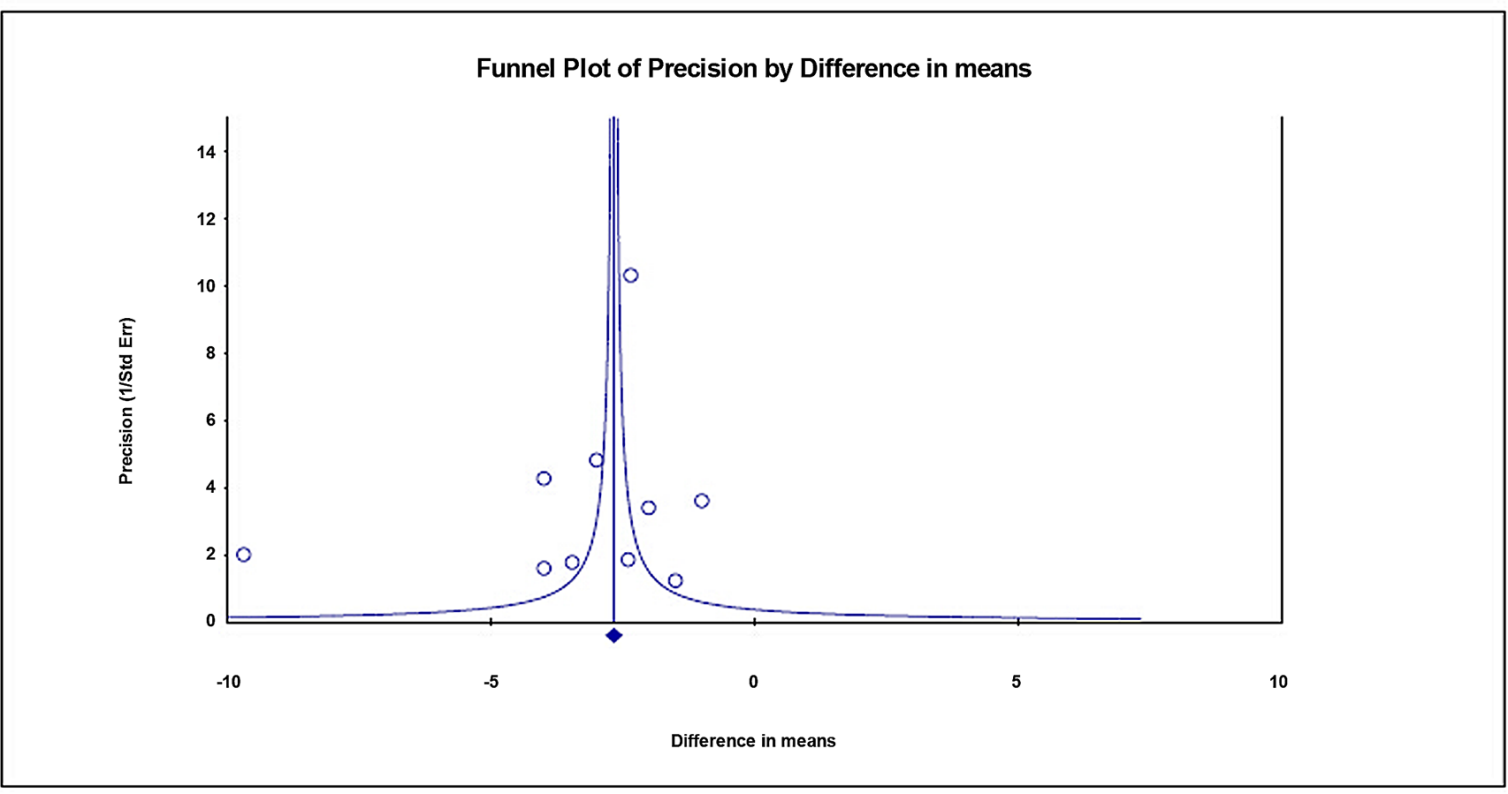
Funnel plot for publication bias for daily pad amount for patients treated with sling.

Sensitivity analysis for daily pad use was performed using the leave-one-out approach in which each study was removed in turn (Fig 6). The direction and magnitude of combined estimates did not vary markedly with the removal of any specific study, indicating that the meta-analysis had good reliability and the data was not overly influenced by any one study. The results via Egger’s test showed there was no publication bias for the findings in regard to daily pad amount for patients treated with sling (t = 1.073, one-tailed, P = 0.157, Fig 7).

## Discussion

This meta-analysis assessed the efficacy of artificial urinary sphincter and sling placement in treating post-prostatectomy incontinence. We found that that both the sling and artificial urinary sphincter significantly decreased the number of pads used per day by about three and increased the quality of life compared with no treatment. In addition, the cure rate (defined as no use of a pad for at least one day) and was around 60%. Intervention resulted in improvement in incontinence (defined as a reduction in the use of pads compared with before intervention) of about 25% (P < 0.001). Our findings indicate that both methods for treating incontinence following a prostatectomy are effective and improve the quality of life for the patient.

This meta-analysis is consistent with prior findings that indicated the sling is an effective method for treating incontinence following prostatectomy surgery. The bone-anchored sling has been assessed in >10 studies with follow-up out to 48 months [6]. Our analysis included 4 studies that investigated the efficacy of bone-anchored sling in treating post-prostatectomy incontinence [22,34–36]. The cure rate in the included studies ranged from 63% to 98% and the quality of life improved post sling surgery for all 4 studies. Retrourethral transobturator slings, such as the AdVance and I Stop TOM slings, have been extensively studied, and in fact, the majority of sling studies in our meta-analysis (n = 16) [12–14,16–21,26,27,29,31–33] investigated the efficacy of this type of sling. The cure rate for the included studies ranged from 9% to 85% and similar to the bone-anchored sling, use of the retrourethral transobturator slings improved the quality of life in all studies. The adjustable suburethral sling has also been evaluated in multiple studies which showed initial success rates of 70%-80% [6]. Our analysis include three studies that investigated this type of sling [15,28,30]. These 3 studies reported a cure rate from 58.3% to 73% and an improvement in quality of life. Other studies with follow-up times of 29 months found an issue with the adjustable suburethral type of sling was serious mechanical and infectious complications, and removal of the sling in about a third of the cases [6].

Our meta-analysis supports multiple prior studies that consistently show efficacy and durability of the artificial urinary sphincter—the gold standard for treating post-prostatectomy urinary incontinence. It has a success rate of >80% regardless of the degree of incontinence [5]. Our analysis showed a cure rate of about 60% and an improvement rate of 25%. The artificial urinary sphincter also has the advantage of being versatile and effective for a wide range of conditions including after failure of other treatments and radiation [46].

A prior systematic review analyzed the continence and complication rates after male slings following first line surgical treatment [47]. Five studies were included that evaluated adults (N = 356) with SUI post-prostatectomy who underwent male sling surgery as the first surgical option for continence recovery and were followed for more than one year. At a median follow-up of 15 months the pooled cure rates for all kinds of slings was 77.4% (95% CI 66.0–85.8). Consistent with our analysis, they found the sling is an effective method for treating incontinence following prostatectomy surgery. However, the authors note that their findings had to be interpreted with caution, due to several limitations, such as the study designs of the included studies, and the limited number of studies included in the meta-analysis. The study did not evaluate complication rate due to lack of data.

There are several drawbacks to our analysis that should be taken under consideration when interpreting the results. Our analysis only include one randomized controlled trial. The vast majority of studies compared outcomes pre- and post-sling or artificial urinary sphincter intervention and did not contain a control group which may impact some of the findings, particularly the subjective outcomes such as quality of life. Issues in regard to study design reflects the quality of the studies available for our meta-analysis. Other study issues include the definition of incontinence, cure rate, and improvement in incontinence varied across studies which may have confounded the results. Currently there is no standardized classification for evaluating outcomes in the evaluation of the efficacy of different methods for treating post-prostatectomy urinary incontinence. Our meta-analysis did not compare the effectiveness of slings to artificial urinary sphincter. Very few studies have compared the effectiveness between different treatments, instead the choice of the treatment was the surgeon’s decision. Well-designed studies are necessary to compare the efficacy across different treatments for incontinence following prostatectomy.

In summary, our meta-analysis found that the use of sling and urinary sphincter are effective in treating post-prostatectomy urinary incontinence, resulting in significantly reduction in the number of pads used per day the patient’s quality of life.

## Supporting information

S1 PRISMA Checklist PRISMA 2009 Checklist. (DOC)
